# Biannual Treatment of Preschool Children with Single Dose Azithromycin to Reduce Mortality: Impact on Azithromycin Resistance in the MORDOR Trial in Tanzania

**DOI:** 10.4269/ajtmh.19-0086

**Published:** 2020-02-17

**Authors:** Evan M. Bloch, Christian L. Coles, Mabula Kasubi, Jerusha Weaver, Zakayo Mrango, Beatriz Munoz, Thomas M. Lietman, Sheila K. West

**Affiliations:** 1 Department of Pathology, Johns Hopkins School of Medicine, Baltimore, Maryland;; 2 Infectious Disease Clinical Research Program, Uniformed University of the Health Sciences, Bethesda, Maryland;; 3 Department of Microbiology, Muhimbili University of Health and Allied Sciences, Dar es Salaam, Tanzania;; 4 Dana Center for Preventive Ophthalmology, Johns Hopkins School of Medicine, Baltimore, Maryland;; 5 National Institute for Medical Research, Kilosa, Tanzania;; 6 Francis I Proctor Foundation, University of California, San Francisco, San Francisco, California

## Abstract

The *Macrolides Oraux pour Réduire les Décès avec un Oeil sur la Résistance* study showed that administration of biannual, single-dose azithromycin to preschool children reduces mortality. We sought to evaluate its impact on azithromycin resistance. Thirty randomly selected communities in Kilosa district, Tanzania, were randomized to receive 6-monthly single-dose azithromycin (∼20 mg/kg) versus placebo treatment of children aged 1–59 months. From each community, 40 children (aged 1–59 months) were randomly selected at baseline, 12 and 24 months. Isolation and resistance testing of *Streptococcus pneumoniae* and *Escherichia coli* were evaluated using nasopharyngeal and rectal swabs, respectively. The carriage prevalence and the proportion of azithromycin-resistant isolates were determined using disk diffusion. At baseline, the characteristics of the randomly selected children were similar by treatment arms. Both at baseline and in annual cross-sectional surveys, rates of *S. pneumoniae* and *E. coli* isolation between treatment arms were similar. The proportions of azithromycin-resistant *S. pneumoniae* isolates in the children in communities treated with azithromycin versus placebo at baseline, 12 months, and 24 months were 26.5% (18.1%; *P* = 0.26), 26.8% (16.5%; *P* = 0.29), and 13.4% (17.0%; *P* = 0.57), respectively. The proportions of azithromycin-resistant *E. coli* isolates at baseline, 12 months, and 24 months in the azithromycin (versus placebo) arms were 14.9% (18.9%; *P* = 0.16), 21.5% (16.6%; *P* = 0.10), and 14.9% (14.7%; *P* = 0.95), respectively. Over the 24 months, the mean treatment coverage for the azithromycin and placebo was 76.9% and 74.8%, respectively (*P* = 0.49). Biannual administration of single-dose azithromycin to children did not appear to result in excess azithromycin resistance in *S. pneumoniae* and *E. coli* isolates over 24 months of follow-up.

## INTRODUCTION

Azithromycin is a versatile antibiotic that is used to treat a variety of infectious diseases that are common in low-resource settings.^1–4^ Mass drug administration (MDA) of azithromycin has also been integral to the ongoing elimination strategy for trachoma.^5^ In the recent *Macrolides Oraux pour Réduire les Décès avec un Oeil sur la Résistance* (MORDOR, clinicaltrials.gov #NCT02048007) trial, biannual administration of single-dose azithromycin to preschool children was shown to reduce all-cause mortality compared with placebo.^6^ If this were to be adopted as a public health intervention, the emergence of bacterial resistance to azithromycin and other macrolides would become a concern. This is particularly the case with *Streptococcus pneumoniae.* In nationwide hospital surveys in the United States, a quarter to almost a third of *S. pneumoniae* isolates have been shown to be resistant to azithromycin.^7,8^ In the Tanzania and Malawi arms of the MORDOR trial, significant protective effects against mortality were not observed. The potential contribution of increased resistance to this lack of effect is unknown.

Studies that have evaluated bacterial resistance to azithromycin following biannual MDA for trachoma control have reported variable findings.^9–14^ In treatment-naïve populations in which entire communities underwent MDA, resistance developed following a single round of azithromycin but was short-lived.^11,15^ However, when MDA was conducted repeatedly, resistance was slow to revert to pretreatment levels following cessation.^16^ In the Tanzanian site of the MORDOR trial, we hypothesized that the proportion of azithromycin-resistant isolates would be greater at 12 and 24 months in children who reside in communities that are randomized to azithromycin as compared with those who reside in communities that are randomized to receive placebo. Therefore, we sought to compare the proportion of azithromycin-resistant isolates of *S. pneumoniae* and *Escherichia coli* from children in the communities randomized to azithromycin versus placebo, comparing the proportions at 12 and 24 months of follow-up.^17–19^

## MATERIALS AND METHODS

### Overview.

A cluster-randomized, placebo-controlled, double-masked clinical trial was conducted in 30 communities in Kilosa district, Tanzania (January 2015–August 2017), as part of the MORDOR trial. This MORDOR morbidity study, which was performed in parallel to the MORDOR mortality trial, sought to evaluate the effect—in children—of biannual, single-dose azithromycin (compared with placebo) on the proportion of azithromycin-resistant isolates of *S. pneumoniae* and *E. coli* that were recovered from nasopharyngeal and rectal swabs, respectively.

### Eligibility.

**Figure 1.**
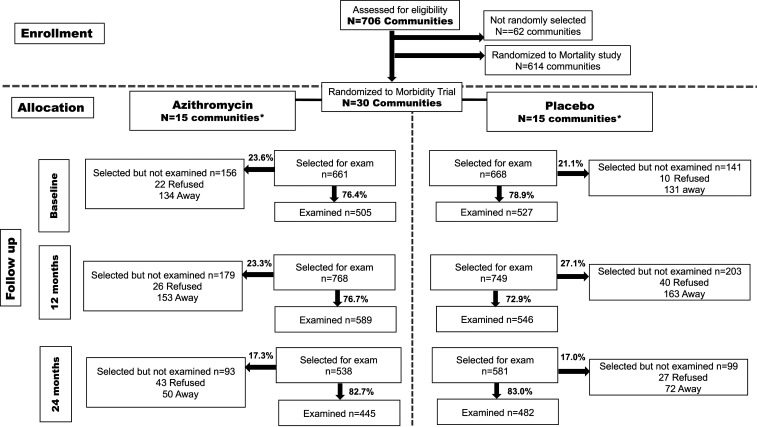
CONSORT diagram. *None of the participating communities were lost to follow-up.

All communities located in Kilosa district that had at least 20 children aged 1–59 months during a baseline census were eligible to participate in the trial (Figure 1). The communities were drawn from the same pool as the MORDOR mortality study.^6^ At each survey time point, all 15 communities in each arm participated. Within each community, 40 children, aged 1 and 59 months, who lived in the randomly selected community at the time of the survey and had a guardian who was capable of providing consent, were randomly selected to participate. Participation rates are shown in Figure 1. If a community had less than 40 eligible children, all the children were selected to participate.

### Randomization and masking.

The lead statistician generated the randomization sequence for the morbidity study, using a series of 6 letters with a 1:1 allocation; this was implemented by the Tanzanian study team. The trial was double-masked such that the treatment assignment was unknown to the participants and study teams. Only the lead statistician who performed the random assignment was unmasked.

#### Intervention.

The intervention was azithromycin (∼20 mg/kg) or placebo (Pfizer, New York, NY); both azithromycin and placebo were supplied in identical containers and were indistinguishable in appearance and taste. The treatments were administered every 6 months for 24 months.

#### Primary outcome.

Proportion of azithromycin-resistant isolates of *S. pneumoniae* and *E. coli* obtained from nasopharyngeal and rectal swabs, respectively, in randomly selected children aged 1–59 months at 12 and 24 months.

### Specimen collection.

In each community, specimens were collected at baseline, 12 months (after two rounds of intervention), and 24 months (after four rounds of intervention). A trained field team collected nasopharyngeal and rectal swabs for culture, before the upcoming intervention. Each nasopharyngeal specimen for *S. pneumoniae* was obtained using a sterile, rayon-tipped swab (Puritan calcium alginate swabs, Fisher Scientific, Hampton, NH). The swab was inserted in media containing skim milk, tryptone, glucose, and glycerin (STGG) and stored at −20°C pending shipment on dry ice to the Muhimbili National Hospital’s microbiology laboratory (MHML), within 1 month of collection. Rectal specimens for *E. coli* were collected using sterile cotton swabs, placed into Amies transport media and stored at −20°C for up to 1-month pending shipment on dry ice to the MHML for isolation and resistance testing. Pilot testing with swabs inoculated with *E. coli* showed that the organisms were still viable after storage at −20°C for approximately 7 weeks.

### Laboratory procedures.

For *S. pneumoniae* isolation, swabs were inoculated onto blood agar containing 5% sheep blood and 5 mg/L gentamicin (Becton Dickinson, Cockeysville, MD) and then incubated at 37°C in 5% CO2 for 18–24 hours. Colonies were confirmed by optochin disk (Taxo, Becton Dickinson) inhibition or bile solubility testing. A pneumococcal reference strain was used for quality control. Culture-positive specimens were subcultured and frozen in STGG at −70°C pending antibiotic susceptibility testing.

The Kirby–Bauer disk diffusion method was used to test *S. pneumoniae* isolates for sensitivity to azithromycin (15 µg disk) on Mueller–Hinton media with 5% lysed sheep blood. Inhibitory zone diameters (ZDs) were used to classify isolates as “sensitive” (ZD > 18 mm), “intermediately resistant” ZD ≥ 14 mm but ≤ 17 mm), or “resistant” (ZD ≤ 13 mm), according to the 2018 Clinical and Laboratory Standards Institute (CLSI) recommendations.^20^

For *E. coli*, swabs were streaked on MacConkey agar and cultured overnight at 37°C. Up to three lactose-fermenting colonies were inoculated into nutrient agar stabs and grown overnight at 37°C followed by storage at room temperature. Indole-positive, citrate-negative isolates were considered to be *E. coli.* Break points to define macrolide resistance in *E. coli* have not been established. Therefore, azithromycin resistance was defined as a ZD < 10 mm; this resistance cutoff was based on a prior study of *E. coli* resistance^21^ and was estimated using a receiver–operator characteristic curve.

### Data management and statistical analyses.

Data were analyzed at each cross-sectional time point, examining the proportion of children with isolates and the proportion of isolates that had resistant organisms, as defined earlier. Descriptive statistics were stratified by the treatment arm. Cross-sectional differences in the proportion of isolates that were resistant were tested initially using logistic models with the arm as the independent predictor. The generalized estimating equations approach, with an independent correlation structure, was used to account for clustering of isolates/resistant isolates within the same community. Data were analyzed with SAS version 9.4 software (SAS, Raleigh, NC). Intermediate and resistant categories were combined and also presented. Coverage with azithromycin/placebo treatment of all children aged 1–59 months in the communities was calculated as follows: the proportion of children treated was calculated for each community, and coverage by the treatment arm was estimated as the mean of the community proportion for communities in that arm. Mean coverages and 95% CIs are reported.

### Sample size calculation.

Given a baseline resistance of 12%,^12^ and an intra-class correlation coefficient of approximately 0.05,^22^ we anticipated approximately 80% power to detect a difference in the proportion of isolates with a resistance of 18% (i.e., comparing 12–30%), assuming at least eight children per community with isolates.

### Ethical review and trial oversight.

Ethical approval was obtained from the Tanzanian National Institute for Medical Research and the institutional review boards of the Johns Hopkins School of Medicine and the University of California San Francisco. Children were included in the study on the basis of documented written informed consent from guardians. The study is registered at clinical trials.gov (NCT02048007). A data and safety monitoring committee provided trial oversight.

### Role of the funding source.

The study sponsor had no role in the study design, the collection, analysis, and interpretation of the data, in the manuscript preparation or the decision to submit the paper for publication.

## RESULTS

At baseline, the demographic and clinical characteristics of the children and rates of isolation of *S. pneumoniae* and *E. coli* between the treatment arms were similar and not significantly different (Table 1).

**Table 1:** Baseline characteristics in randomly selected children by the community treatment arm

Characteristic	Arm		*P*-value
	Azithromycin, *N* = 505	Placebo, *N* = 527	
Age (months), mean (SD)	27.3 (16.1)	28.1 (15.9)	0.36
Female, *n* (%)	246 (48.7)	263 (49.9)	0.69
Used drugs to treat infection previous 14 days, *n* (%)	117 (23.2)	173 (32.9)	0.06
Child is coughing, *n* (%)	177 (35.0)	172 (32.8)	0.63
Child has difficulty breathing, *n* (%)	8 (1.6)	4 (0.8)	0.21
Respiratory rate (breaths/minute), mean (SD)	37.7 (4.1)	37.6 (3.5)	0.66
Child has diarrhea, *n* (%)	46 (9.1)	57 (10.8)	0.46
Child has fever, *n* (%)	35 (6.9)	35 (6.7)	0.88
Laboratory results			
*Streptococcus pneumoniae* isolated, *n* (%)	219 (43.5)	226 (43.0)	0.90
*Escherichia coli* isolated, *n* (%)	310 (61.6)	324 (61.5)	0.99

The average treatment coverage in the communities that received azithromycin was 76.9%. Average coverage of the communities was significantly different between the two arms only at the 12-month treatment round, where there was higher coverage in communities in the azithromycin arm (77.2% versus 64.8%) (Table 2).

**Table 2:** Treatment coverage in 30 communities randomized to azithromycin vs. placebo

Period	Average community coverage*		*P*-value
	Azithromycin, mean (95% CI)	Placebo, mean (95% CI)	
Baseline	78.1 (70.0–86.6)	79.6 (72.5–86.6)	0.78
6 months	75.0 (67.0–83.0)	71.7 (64.4–79.1)	0.21
12 months	77.2 (71.2–82.6)	64.8 (51.2–75.8)	0.04
18 months	77.4 (72.1–82.8)	76.2 (69.4–83.0)	0.76

* Estimated as the mean of the proportion treated in each community.

At 24 months, over half the children in both arms took the drug as randomized for at least three of four of treatment cycles (Table 3). In the annual cross-sectional surveys, rates of *S. pneumoniae* and *E. coli* carriage between treatment and placebo arms were similar. However, rates of isolation declined overtime (Table 3). The respective rates of *E. coli* isolation in the children in the azithromycin arm were 61.6%, 39.6%, and 58.9% compared with 61.5%, 43%, and 43.2% in the placebo arm.

**Table 3:** Characteristics of randomly selected children at 12 and 24 months (post two and four rounds of treatment)

Characteristic	12-month arm		*P*-value	24-month arm		*P*-value
	Azithromycin, *N* = 589	Placebo, *N* = 546		Azithromycin, *N* = 445	Placebo, *N* = 482	
Age (months), mean (SD)	25.5 (14.1)	26.1 (15.5)	0.51	29.7 (16.1)	26.6 (16.6)	0.03
Female, *n* (%)	297 (50.4)	261 (47.8)	0.30	226 (50.8)	228 (47.3)	0.34
Laboratory results						
*Streptococcus pneumoniae* isolated, *n* (%)	97 (16.5)	97 (17.8)	0.78	67 (15.1)	53 (11.0)	0.26
*Escherichia coli* isolated, *n* (%)	233 (39.6)	235 (43.0)	0.54	262 (58.9)	272 (43.2)	0.71
Number of previous visits during which the child received the study drug						
None, *n* (%)	102 (17.3)	95 (17.4)	0.97	32 (7.2)	44 (9.1)	0.33
One, *n* (%)	160 (27.2)	146 (26.7)		49 (11.0)	54 (11.2)	
Two, *n* (%)	327 (55.5)	305 (55.9)		79 (17.8)	88 (18.2)	
Three, *n* (%)	–	–	–	103 (23.1)	134 (27.7)	
Four, *n* (%)	–	–	–	182 (40.9)	163 (33.8)	

An excess in the proportion of azithromycin-resistant *S. pneumoniae* and *E. coli* isolates was not found at baseline, 12 months, or 24 months (Table 4): the respective proportions of azithromycin-resistant *S. pneumoniae* isolates in azithromycin versus placebo arms at baseline, 12, and 24 months were 26.5% versus 18.1% (*P* = 0.26), 26.8% versus 16.5% (*P* = 0.29), and 13.4% versus 17.0% (*P* = 0.57). The proportions of azithromycin-resistant isolates of *E. coli* at baseline, 12, and 24 months in the azithromycin versus placebo arms were 14.9% versus 18.9% (*P* = 0.16), 21.5% versus 16.6% (*P* = 0.10), and 14.9% versus 14.7% (*P* = 0.95), respectively (Table 4).

**Table 4:** Percentage of *Streptococcus pneumoniae* and *Escherichia coli* isolates resistant to azithromycin by the study arm and time of survey

Survey time	Characteristic	Arm		*P*-value*
		Azithromycin	Placebo	
		Proportion of resistant isolates (95% CI)*	Proportion of resistant isolates (95% CI)*	
Baseline	*S. pneumonia* (*N* = 219, 226)†	–	–	–
	Intermediate	9.1	6.6	
	Resistant	17.4	11.5	
	Intermediate/resistant	26.5 (16.5–39.6)	18.1 (10.9–28.6)	0.26
	*E. coli* resistance (*N* = 309, 323)†	14.9 (11.6–18.9)	18.9 (14.9–23.6)	0.16
12 months	*S. pneumonia* (*N* = 97, 97)†	–	–	–
	Intermediate	8.2	2.1	
	Resistant	18.6	14.4	
	Intermediate/resistant	26.8 (13.9–45.4)	16.5 (8.1–30.7)	0.29
	*E. coli* resistance (*N* = 233, 235)†	21.5 (17.0–26.7)	16.6 (13.5–20.3)	0.10
24 months	*S. pneumonia* (*N* = 67, 53)†			
	Intermediate	0.0	3.8	
	Resistant	13.4	13.2	
	Intermediate/resistant	13.4 (8.4–20.8)	17.0 (8.2–31.8)	0.57
	*E. coli* resistance (*N* = 262, 272)†	14.9 (10.8–20.0)	14.7 (10.9–20.1)	0.95

* Using the generalized estimated equation approach with an independent correlation structure to account for clustering within the same community.

† Number of isolates (azithromycin arm and placebo arm).

## DISCUSSION

The aggregate findings from the MORDOR trial showed that targeted biannual administration of single-dose azithromycin reduces mortality in children younger than 5 years.^6^ However, a reduction in mortality was not seen in the Tanzanian (also Malawian) arm of the MORDOR trial. This raised the question as to whether emergence of antibiotic resistance could have offset any potential morbidity or mortality benefit in these sites. Concern over azithromycin resistance is not limited to bacteria for which it is known to be active (e.g., *S. pneumoniae*), rather antibiotic selection pressure can extend to nontarget bacteria such as *E. coli*.^21^ This is well established, particularly in poor-resource settings.^23,24^ Commensal bacteria can also serve as important reservoirs for resistant bacteria.^25,26^ We hypothesized that children in the villages randomized to receive azithromycin would have significantly more isolates resistant to azithromycin than children in the villages randomized to receive the placebo. However, neither an excess in the proportion of azithromycin-resistant *S. pneumoniae* and *E. coli* isolates nor an increase in resistance overtime were found at either 12 or 24 months.

Most studies that have reported on azithromycin resistance in *S. pneumoniae* isolates have been conducted in the context of trachoma in which all residents in the communities have undergone azithromycin treatment, although resistance testing may be confined to children. Most studies show an increase in azithromycin-resistant strains of *S. pneumoniae* immediately following MDA. In treatment naïve populations, a single round of mass treatment is followed by an increase in resistance that subsides after a few months.^9^ One study in Tanzania observed nearly absent resistance at 6 months following a single round of MDA.^9^ However, when multiple rounds of MDA have been given, the decline is not as rapid.^10,13^ One study evaluated azithromycin resistance in fecal *E. coli* in eight communities following a single round of MDA in a district that had previously undergone multiple rounds of MDA; although carriage prevalence of resistant strains at baseline was similar between children in newly treated and untreated communities, the carriage of resistant isolates increased from 21% to 61% at 1 month and was still elevated (31%) at 6 months in the newly treated communities.^21^

Studies of antibiotic resistance where administration of azithromycin has been targeted to children are few. In one small randomized trial in Ethiopia, children aged 1–10 years underwent 3-monthly single-dose azithromycin versus no treatment for 1 year.^12^ The baseline mean proportion of azithromycin-resistant isolates of *S. pneumoniae* in the treatment arm was 6.3%, which increased to 62.3% at 12 months. Although baseline resistance was not reported in the untreated communities, 11.6% of *S. pneumoniae* isolates were resistant at 12 months.^12^

Several possible explanations were considered as to why we did not find an increase in resistant isolates. The most likely is that unlike the trachoma trials that treat everyone in the community, we only treated children aged 1–59 months, who represent a small (15% or less) fraction of the community. This leaves a larger population pool with susceptible strains that are available to repopulate the nasopharynx of the treated children. Not only was there no increase in resistance by treatment assignment but also the proportion of azithromycin-resistant *S. pneumoniae* isolates did not increase from baseline, and, if anything, the point estimate decreased by 24 months, following four rounds of drug administration. Had there been a lasting increase in resistance (i.e., as a result of multiple rounds of azithromycin treatment of children in those communities), the 24-month survey would have been the most informative.

We acknowledge that with follow-up visits at 1 year and 2 years, we were not able to detect evidence of a transient increase in resistance that might have occurred immediately following treatment cycles. The effect of any such increase is uncertain. If such transient increases led to increased disease during that time, we would have expected to see an increase in deaths in the azithromycin arm during that small window. However, another study has shown that there were fewer deaths in the period immediately following azithromycin administration.^27^ Further, one would expect that if resistance were to increase, it would be most evident following multiple doses of azithromycin. Although we cannot rule out transient resistance (i.e., observable at times less than 12 months), our findings suggest that the children were recolonized by 12 months to approximate the community prevalence of resistance. As such, treatment of this small subgroup of children compared with the general population may have been insufficient to alter the overall community pattern of resistance. Low rates of resistance following targeted administration of azithromycin was also observed in a study in Nepal,^28^ Our findings could be due to incomplete coverage of children with azithromycin. The mean coverage of communities in the azithromycin arm was 75–78%; thus, there were some children in the 1- to 59-month age range, most with presumably susceptible strains, which did not contribute to any increase in resistance. Our study, a cluster-randomized trial, was designed to detect an increase in resistance in children living in communities that were randomized to receive either azithromycin or placebo, rather than to evaluate resistance in individual children who had been treated or not treated. From previous research, it is unclear what level of community coverage is needed to drive the increase and durability of resistance, in part because coverage has not been reported or has been reportedly very high in previous studies.

Third, our surveys were cross-sectional representations of resistance in children in the participating communities. Given random sampling of children at the time of survey (with no requirement of previous residency in the community), it is possible that some surveyed children did not receive prior doses of azithromycin, which could have lowered the rates of resistance in the azithromycin arm. Nonetheless, more than half of the sample had received at least three of the four doses at 24 months, suggesting that the composition of the sample was not a major contributing factor.

The study medications were completely masked to the study team and participants, and there is no evidence this was breeched. Therefore, it is unlikely that the results reflect inadvertent receipt of azithromycin in the control communities or vice versa.

A significant limitation to the study is the decline in recovery rates of both organisms (*S. pneumoniae* and *E. coli*) overtime. In the case of *E. coli*, one would expect isolation rates of 90–100%^29^; instead, even at baseline, recovery rates were unexpectedly low (62%). This likely lessened the study’s power to detect a difference in prevalence of resistance between the intervention and placebo arms. Nonetheless, the point estimates in the treatment and placebo arms were not markedly dissimilar, offering some plausibility to the findings. In the case of *S. pneumoniae*, the low recovery may be attributed—in part—to pneumococcal vaccination in Kilosa in the year following initiation of our study. Vaccination will affect carriage rates of those *S. pneumoniae* serotypes covered by the conjugate vaccine in use; it will also alter nasopharyngeal colonization both with non-vaccine serotypes and competing flora.^30^ In the absence of serotyping, it is difficult to quantify that effect. Regional studies of *S. pneumoniae* vaccine coverage before vaccination have shown variable (23–42%) rates of recovered isolates to be 10-valent pneumococcal conjugate vaccine (PCV10) vaccine serotypes.^31,32^ In Kenya, Hammitt et al.^33^ evaluated annual cross-sectional *S. pneumoniae* carriage in the 2 years before and after incorporation of the PCV10 into the national immunization program. Among children younger than 5 years, the baseline carriage rates for vaccine and non-vaccine *S. pneumoniae* serotype were 34% and, 41% respectively. Subsequent to PCV10 vaccination, the carriage rates were 13% and 57%, respectively.^33^ Serotyping was not performed in our study and might have offered an insight into the impact of the pneumococcal vaccine on the findings.

Transportation and storage may also have affected recovery rates, despite strict cold chain maintenance, spanning collection, the use of guarded freezers, active monitoring of generators, and transportation of specimens on dry ice to the laboratory at Muhimbili. Nonetheless, we acknowledge the potential impact of transport and storage on labile organisms such as *S. pneumoniae*. Of note, any external factor that may have impacted the isolation rate had a similar effect in both the intervention and control communities so that an elevated level of resistance—if present—should have been detected even with fewer isolates. Furthermore, we used the most current CLSI guidelines to categorize resistance^20^; differences in break points across guidelines can impact the analysis. Differences in the definition of resistance also account for changes in the reporting; specifically, previous publication of our baseline resistance did not combine intermediate with resistant strains.^16^ Finally, there is a complex interplay between antibiotic resistance and bacterial fitness.^34,35^ Although development of resistance should confer survival advantage in the presence of the cognate drug, this could also come at the cost of a competitive fitness disadvantage during storage, thereby impacting the recovery rates. We note, however, that the isolation rate was not lower in the children in the azithromycin arm, which likely would have been the case if there were more resistant strains that could not be recovered.

In conclusion, a randomized controlled trial in Tanzania did not show an increase in azithromycin resistance within *S. pneumoniae* and *E. coli* isolates after four biannual rounds of treatment. It is important to emphasize that our reporting was confined to only two organisms. Despite their clinical importance, *S. pneumoniae* and *E. coli* fail to represent the spectrum of organisms that might be susceptible to selection pressure. Although azithromycin resistance in *S. pneumoniae* or *E. coli* does not appear to account for lack of efficacy against child mortality for this site,^6^ the findings should be interpreted together with the acknowledged limitations of this study.
